# Genetic diversity of durum wheat (*Triticum turgidum* ssp. durum) to mitigate abiotic stress: Drought, heat, and their combination

**DOI:** 10.1371/journal.pone.0301018

**Published:** 2024-04-04

**Authors:** Latifa Chaouachi, Miriam Marín-Sanz, Francisco Barro, Chahine Karmous

**Affiliations:** 1 Laboratory of Genetics and Cereal Breeding (LR14 AGR01), National Institute of Agronomy of Tunisia, Carthage University, Carthage, Tunisia; 2 Department of Plant Breeding, Institute for Sustainable Agriculture-Spanish National Research Council (IAS-CSIC), Córdoba, Spain; ICAR Indian Institute of Wheat and Barley Research, INDIA

## Abstract

Drought and heat are the main abiotic constraints affecting durum wheat production. This study aimed to screen for tolerance to drought, heat, and combined stresses in durum wheat, at the juvenile stage under controlled conditions. Five durum wheat genotypes, including four landraces and one improved genotype, were used to test their tolerance to abiotic stress. After 15 days of growing, treatments were applied as three drought levels (100, 50, and 25% field capacity (FC)), three heat stress levels (24, 30, and 35°C), and three combined treatments (100% FC at 24°C, 50% FC at 30°C and 25% FC at 35°C). The screening was performed using a set of morpho-physiological, and biochemical traits. The results showed that the tested stresses significantly affect all measured parameters. The dry matter content (DM) decreased by 37.1% under heat stress (35°C), by 37.3% under severe drought stress (25% FC), and by 53.2% under severe combined stress (25% FC at 35°C). Correlation analyses of drought and heat stress confirmed that aerial part length, dry matter content, hydrogen peroxide content, catalase, and Glutathione peroxidase activities could be efficient screening criteria for both stresses. The principal component analysis (PCA) showed that only the landrace Aouija tolerated the three studied stresses, while Biskri and Hedhba genotypes were tolerant to drought and heat stresses and showed the same sensitivity under combined stress. Nevertheless, improved genotype Karim and the landrace Hmira were the most affected genotypes by drought, against a minimum growth for the Hmira genotype under heat stress. The results showed that combined drought and heat stresses had a more pronounced impact than simple effects. In addition, the tolerance of durum wheat to drought and heat stresses involves several adjustments of morpho-physiological and biochemical responses, which are proportional to the stress intensity.

## Introduction

Durum wheat is one of the most common cereal crops in the Mediterranean basin and the tenth most cultivated species worldwide. Despite accounting for only 5% of global wheat production, this wheat species is a key commodity for many worldwide areas such as the Mediterranean basin, North America, northern Mexico, and sub-Saharan Africa [1]. Moreover, durum wheat production is highly dependent on climate change. Indeed, drought and heat stresses are two major factors limiting wheat yield and productivity, leading to irreversible damage [2]. Both abiotic stresses cause reductions in the number and weight of grains and thus significant losses in yield and grain quality [3].

In recent decades, one of the clearest signs of climate change is the increase in global average temperature. Global warming caused by greenhouse gases is causing extreme weather events including sirocco winds and heat waves affecting all parts of the globe [4]. By 2025, climate scenarios predict that two-thirds of arable land will be lost due to rising temperatures [5].

Heat stress is an important factor limiting crop productivity, particularly when extreme temperatures occur at plant growth stages as for cereal [6]. Heat stress is becoming one of the most threatening stresses for agriculture productivity and the sustainability of a set of species. In fact, during the last thirty years, the earth’s temperature increased by 0.2°C. Each decade, a warming of 1.4 to 5.8°C is predicted during the 21^st^ century [7]. Many studies reported that high temperatures above the optimum will negatively affect both crop yield and quality [8,9]. Therefore, robust wheat varieties that can acclimate to adverse climate conditions are becoming a priority for crop breeding [10]. Since high temperatures led to morphological, physiological, and biochemical changes, plants have developed a set of tolerance mechanisms as short-term mechanisms of avoidance or acclimation and/or long-term mechanisms of phenological and morphological adaptations [11,12]. Thermotolerance is one of the heat-resistant mechanisms induced to protect the photosynthetic apparatus under heat stress [13,14]. In addition, plants can avoid heat wave damage by increasing both stomatal conductance and transpiration rates, thereby enhancing leaf cooling [15]. However, the variation among wheat genotypes in terms of their photosynthetic thermal acclimation under high temperatures is still unclear [10]. On the other hand, water stress limits plant growth and production, can affect seed germination, and lead to late and incomplete emergence as it can be the cause of a reduction in the number of ears per unit area [16]. Also, among the drawbacks of water scarcity in durum wheat cultivation is the reduction of plant height, and hormonal imbalance [17,18]. The effect of abiotic stress is dependent on its intensity, frequency, and duration, as well as on cropped varieties and growth stages [19].

As plants have developed several adaptive physiological and metabolic mechanisms, the sensitivity of genotypes to stress episodes showed huge variability [20,21]. Many studies focused on crop physiological adjustment to specific stresses such as heat [11,22] and drought [23,24]. However, crops respond differently to combined abiotic stress [25,26] occurring simultaneously which causes complex responses and interconnected signaling pathways that could conflict with each other [27]. Under heat stress, plants with adequate water supply keep their stomata open to enhance leaf cooling through transpiration, while under water scarcity plants close their stomata to avoid excess water loss resulting in increased leaf temperature [13]. In tobacco and wheat, combined drought and heat stress have more severe effects on the rates of growth and development, as well as photosynthetic rate, stomatal conductance, and chlorophyll fluorescence, compared to the effect of individual stress [28,29]. Under combined stress, the plants’ response is affected by the most intense stress type [30].

All abiotic stressors, such as heat or water, lead to oxidative stress. Indeed, oxidative stress results from an imbalance between Reactive Oxygen Species (ROS) and antioxidant defenses. ROSs play a dual role in plant response to abiotic stresses. Indeed, they are both toxic by-products of stress metabolism but also important molecules in the transduction of cellular signals [31]. Indeed, at low concentrations, hydrogen peroxide (H_2_O_2_) acts as a signal molecule involved in the regulation of biological/physiological processes (photosynthetic functions, cell cycle, growth and development, plant responses to biotic and abiotic stresses), however, high H_2_O_2_ concentration leads to the appearance of oxidative stress which can end in cell death. In addition, excess ROS production in response to drought and heat stresses can cause damage to proteins, carbohydrates, lipids, and nucleic acids [32]. Moreover, various ROS transmitted to the mitochondrion play a role in the adaptive response of the mitochondrial redox state, especially for the reduction state of respiratory pathways. The redox signals will be transmitted to the nucleus to regulate plant growth and development [33].

The H_2_O_2_ signaling pathway in response to water stress also promotes the accumulation of several cell protectors that can act directly or indirectly in the regulation of the cellular redox state such as enzymatic antioxidants [31]. These antioxidants can be categorized into preventative antioxidants, scavenger antioxidants, and de novo and repair antioxidants. Preventative antioxidants act as the first line of defense in the cell, preventing the formation of ROS [34]. The ability to regulate the antioxidant enzyme activities is the main mechanism of adaptation to water stress [35]. Enzymatic antioxidants (Superoxide dismutase (SOD), glutathione reductase (GR) is considered the first line of defense against oxidative stress. The main detoxification enzymes are GR, catalase (CAT), Glutathione Peroxidase (GPX), superoxide dismutase (SOD), ascorbate peroxidase (APX), glutathione peroxidase (GPX), and peroxiredoxin (Prx) [36]. Thioredoxins (Trx) are small proteins able to catalyse thiol-disulfide interchange and are involved in the regulation of the redox environment in the cell [37]. At the start of the reaction, a primary ROS which is superoxide anion (O_2_-) can be formed by the one-electron reduction of molecular oxygen [38]. The superoxide anion (O_2_^-^) is dismutated by SOD to H_2_O_2_ which is detoxified by CAT [39]. Once the O_2_^-^ is formed in the presence of H_2_O_2_, it becomes inevitable. Further reactions may lead to the formation of hydroxyl radicals (HO^-^) [38]. In fact, antioxidant proteins protect the damage of ROS leaking from peroxisomes [40,41].

GPX is a heme-containing enzyme that removes excess amounts of H_2_O_2_ under normal and stressful conditions [42]. In addition, several previous studies have shown that GPXs play an essential role in plant response to abiotic stresses such as heat, cold, drought, salt, and oxidative stress [43–46]. CAT is a tetrameric enzyme, involved in the cell’s defenses against oxidative stress by eliminating ROS [47]. Catalase activity is increased when the level of oxidative stress is high, or the amount of glutathione peroxidase is low [48].

Among the latter, phenolic acids are the major subclass of polyphenols participating in the scavenging of ROS in cereals [49]. The content and composition of phenolic acids may vary in the durum wheat germplasm [50]. The extent of variation for phenolic acids is up to 3.6-fold [51–53], and it is influenced by both the genotype and environmental factors [51–53].

This research is a primordial step in a genetic improvement and selection program to determine the physiological processes related to the plant’s response to abiotic stress. The study of these mechanisms is a determinant of genotypic tolerance to stress in durum wheat [54]. Nevertheless, few studies have focused on the combined effects of abiotic stresses and the majority have focused on the single effects of stresses.

Specifically, this study aimed to (i) determine genotypic variation and its interactions with individual water/heat and combined treatments, (ii) select genotypes with high potential of tolerance under stressful conditions and (iii) identify traits conferring heat and drought tolerance.

## Materials and methods

### Plant material and growth conditions

The used plant material is constituted of five durum wheat (*Triticum turgidum* ssp. *durum*) genotypes composed of four landraces genotypes (Hmira, Biskri, Hedhba, and Aouija) from the national gene bank (BNG) and an improved variety (Karim). Three assays were conducted on (i) drought, (ii) heat stress, and (iii) the interaction of two stresses at the juvenile growth stage (Z14) according to Zadok’s growth scale [55].

**Fig 1:**
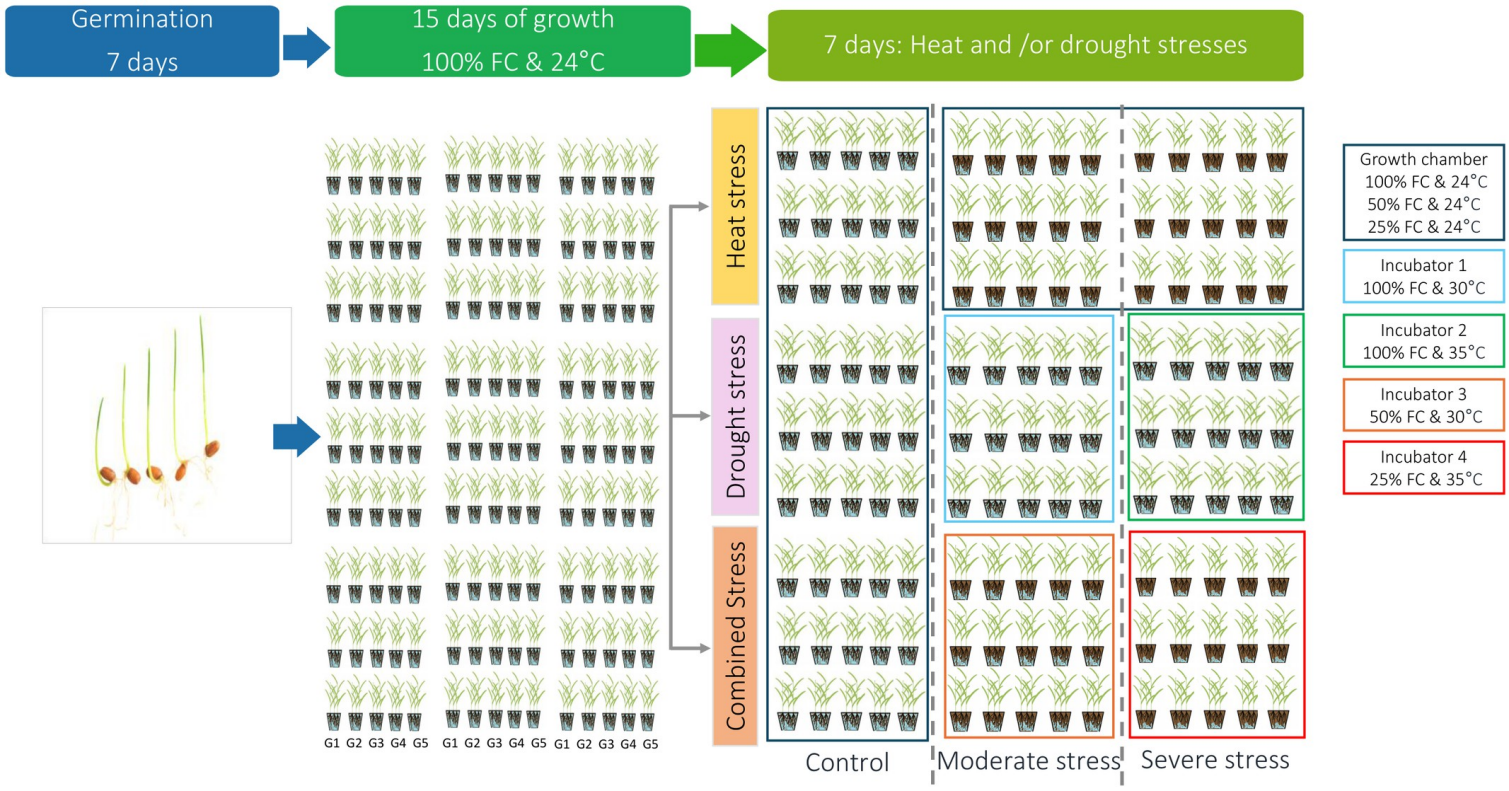
Representative illustration of the experimental setup deployed in the culture chamber and four incubators for the study of the effect of water stress/or thermal stress on the growth of five durum wheat genotypes: G1: Aouija, G2: Biskri, G3: Hedhba, G4: Karim, G5: Hmira.

The grains were disinfected with 20% bleach for 10 min., then rinsed three times with distilled water, and then they were kept for 45 min. in distilled water before sowing with 3 plants per pot (2 L) containing a mixture of peat: perlite (2:1; V/V). The experimental assays were arranged as a completely randomized design with a total number of pots of 135 pot at the rate of 3 pots/genotype/treatment. The plants were grown under controlled conditions with a temperature of 21 ± 2°C (night/day), a photoperiod of 16h, and an average humidity of 60%. After 15 days of growing, three drought treatments (100, 50, and 25% of soil capacity (FC)), three heat stress treatments (24, 30, and 35°C), and three combined treatments (100% FC at 24°C, 50% FC at 30°C and 25% FC at 35°C) were assayed. The control plants of the 3 stresses as well as the drought stress treatments (100, 50, and 25% FC) were conducted in the same growth chamber. The heat and the combined stress (Heat + Drought) stresses were assayed in four different incubators as a stress treatment level. The sampling was set up 7 days after stress application (Fig 1).

### Morpho-physiological traits

A set of morpho-physiological traits were assessed. The aerial part length (APL) was measured by a caliper.

The dry matter (DM) rate (%) was recorded for all aerial plant parts. After measuring the initial fresh weight (FW), they were put in an oven at 80°C for 48 hours. The obtained material was weighted as dry weight (DW). The DM rate (%) was obtained according to the present formula:

$$DM\;\left(\text{\%}\right)=\frac{FW}{DW}\times 100$$ (1)

### Biochemical traits

The H_2_O_2_ content was determined by spectrophotometry according to [56]. Indeed, 10^−4^ kg of fresh plant material was ground in 10^−3^ L of a 0.1% trichloroacetic acid (TCA) solution. The ground material was then centrifuged at 12,000 rpm for 15 min. at 4°C. Then, a volume of 0.5.10^−3^ L of the supernatant was incubated in the presence of 10^−3^ L of potassium iodide (KI) (1M) was added to 0.5.10^−3^ L of potassium phosphate buffer (KH_2_PO_4_/K_2_HPO_4_; 10^−2^ M; pH = 7). The mixture was then homogenized and incubated for 5 min. The optical density was determined at 390.10^−9^ m. The next biochemical measure was the total phenolic compounds or polyphenols (Ph.C), which were determined according to the method of [57]. Indeed 5.10^−4^ kg of plant material was ground in 2.10^−3^ L of 80% (v/v) methanol. The ground material obtained was centrifuged at 1,000 rpm for 10 min. A reaction mixture was formed by 10^−4^ L of the supernatant, 1.75 10^−3^ L of sterile water, 25.10^−6^ L of Folin-Cicalteu reagent, and 50.10^−6^ L of sodium carbonate Na_2_CO_3_ (20%). The reaction mixture was then incubated in a water bath at 40°C for 30 min. After cooling, the absorbance was measured at 760.10^−9^ m.

### Antioxidant enzymes activity

The activity of two antioxidant enzymes was measured: GPX and CAT. The activity of GPX was measured according to the method reported by [58]. Thus, 5.10^−4^ kg of fresh plant material was ground in a mortar containing 5.10^−3^ L of a phosphate buffer (5.10^−2^ M; pH = 6.5). The ground material obtained was then centrifuged at 12,000 rpm for 20 min. at 4°C. The enzymatic activities as well as the protein contents were assayed on this extract. The measurement was carried out in a volume of 3.10^−3^ L containing the phosphate buffer (5. 10^−2^ M; pH = 6.5), guaiacol (5. 10^−2^ M), H_2_O_2_ (2%), and 10^−4^ L of enzymatic extract. Enzyme activity was monitored by spectrophotometry at 470.10^−9^ m. The catalase activity was determined spectrophotometrically at 240.10^−9^ m, according to the method described by [59]. Thus, 100 mg of fresh plant material was ground in a mortar with 1.5. 10^−3^ L of phosphate extraction buffer (5. 10^−2^ M; pH = 7). The ground material was then centrifuged at 12,000 rpm for 5 min. at 4°C. The enzymatic activities as well as the protein contents were assayed on this extract. For a final volume of 10^−3^ L, a reaction mixture consisting of 50.10^−6^ L of supernatant and 9.5. 10^−4^ L of H_2_O_2_ reagent (2. 10^−2^ M) was prepared. Absorbance was monitored at 240.10^−9^ m.

### Statistical analysis

For statistical analysis, R software v.3.6.1 was used [60]. The effects of drought and/or heat stresses treatments on the studied genotypes were determined by a multivariate analysis of variance (MANOVA) test. A post-hoc analysis was carried out using Tukey’s multiple comparison test. All measured variables were used for the principal component analysis (PCA) which was performed using the FactoMineR [61] and Factoextra [62] R libraries. The heatmap and dendrogram construction were performed with the heatmap R library [63].

## Results

### Effect of abiotic stress (drought, heat, and combined stress) on durum wheat growth

#### Variation of all measured traits

The MANOVA showed that durum wheat genotypes (G), as well as drought stress, heat, and combined (Heat + Drought) treatments (T), significantly affected all the measured traits (*P*< 0.001) (S1 Table).

**Fig 2:**
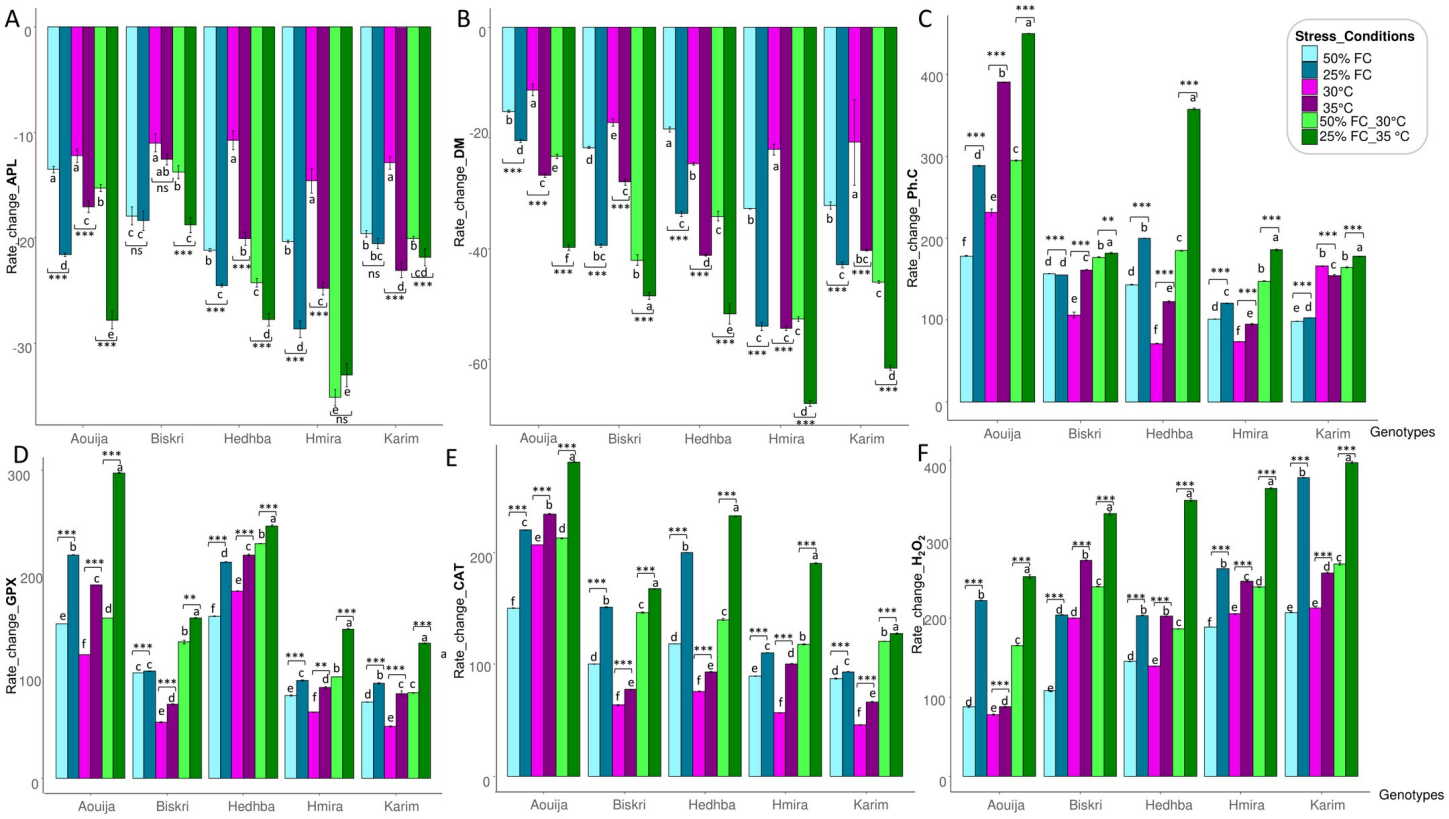
Rate change of six measured variables of five durum wheat genotypes subjected to two levels of drought stress (50% FC and 25% FC), heat stress (30°C and 35°C), and combined stress (50% FC at 30°C and 25% FC at 35°C), evaluated in pots at the juvenile stage (Z14). Such as A) Aerial part length (APL), B) Dry matter rate (DM), C) H_2_O_2_ content (H_2_O_2_), D) Phenolic compounds content (Ph.C), E) GPX activity (GPX), F) CAT activity (CAT), *, *P*<0.05; **, *P*<0.01; ***, *P*<0.001; NS: Not significant.

Fig 2A showed that APL was more affected by drought stress than heat stress treatments, in almost all the genotypes. In fact, under severe drought stress conditions, all durum wheat genotypes showed a decrease in APL, being the lowest decrease in APL observed in the Biskri genotype with 18.4%. However, the most important decrease of APL was observed in the genotype Hmira with 28.7% (Fig 2A). In addition, increasing drought stress induced a decrease in APL with 18.5% under moderate stress (50% FC) and 22.8% under severe stress (25% FC) compared to the control (Fig 2A). Under heat stress, all studied genotypes showed a decrease in APL. Indeed, the temperature of 30°C was at the origin of an average reduction of 12.3% compared to the control (24°C). The greatest decrease was observed in the Hmira genotype (14.6%) compared to the control. However, the least significant decrease was noted in the two genotypes Hedhba and Biskri with 10.8% and 11%, respectively. On the other hand, the temperature of 35°C induced an average decrease in APL of 19.3% compared to the control.

The most important decrease was noted in Hmira and Karim with 23.9% and 24.1%, respectively (Fig 2A). The APL tended to increase under the increasing effect of combined stress. The maximum increase in APL was recorded under severe stress (25% FC at 35°C) with 25.5% and against 21.01% under moderate stress (50% FC at 30°C), compared to the control treatments. The results showed that the landrace Hmira had the highest decrease in APL under the two combined stresses of 50% FC at 30°C as well as under 25% FC at 35°C (Fig 2A).

The dry matter rate (DM) was decreased with the imposition of abiotic stresses. Durum wheat plants under either of the heat, drought, and drought + heat stress conditions promptly decreased (DM) as compared with the controlled treatments (S1 Table).

As shown in Fig 2B, combined heat and drought stress induced more severe changes than the individual effect of drought and heat stress. Thus, DM decreased under heat stress by 37.1% (35°C), by 37.3% under severe drought stress (25% FC), and by 53.2% under severe combined stress (25% FC at 35°C).

The extent of oxidative stress was measured through H_2_O_2_ content. Moreover, oxidative stress was observed mainly under water stress compared to heat stress (S1 Table). The results showed that under drought stress, the H_2_O_2_ content increased by 145.4% and 253.5% under 50 and 25% FC, respectively compared to the control. Under 50% FC, the greatest increase was observed in the genotype Karim followed by Hmira with 207.3% and 188.8%, respectively. However, the least significant decrease was noted for the landrace Aouija with 88% (Fig 2C). Under severe stress (25% FC), the greatest increase was also noted in the improved variety Karim with 377.3% compared to the control (Fig 2C). Under heat stress, the same increase trends were observed with increasing stress levels and for all tested genotypes. The increase in H_2_O_2_ was 164.6% and 210% at 30°C and 35°C, respectively, compared to the control (24°C). Under severe stress, the highest H_2_O_2_ content was observed in both Biskri and Karim genotypes with 275% and 255.6%, respectively, against a minimum in Aouija (88%) (Fig 2C). The H_2_O_2_ content tended to increase under the increasing effect of combined stress. The maximum increase in H_2_O_2_ was recorded under severe stress (25% FC at 35°C) with 337.5% and against 218.9% under moderate stress (50% FC at 30°C), compared to the control. The results showed that the improved variety Karim has the highest H_2_O_2_ content under the two combined stresses of 50% FC at 30°C as well as under 25% FC at 35°C (Fig 2C).

Under drought stress, the studied genotypes tended to increase the leaf concentration of phenolic compounds (S1 Table). Under moderate stress (50% FC), genotypes showed an average increase in polyphenol concentration of 137.6% compared to the control treatment (100% FC). This increase is well marked in the genotype Aouija with 178.5%. Similarly, under severe stress (25% FC), all the genotypes showed an average increase in phenolic compounds of 176.2% compared to the control. The greatest increase was observed in landrace Aouija (288.5%) against a minimum in improved variety Karim (102.8%) (Fig 2D). Under heat stress, durum wheat genotypes tended to show an increase in phenolic compounds. Indeed, under moderate heat stress (30°C), an increase of 132.6% was observed in the genotypes compared to the control. The greatest increase was observed in Aouija with 218% (Fig 2D). Under severe heat stress (35°C), all genotypes showed an average increase of 189.5% compared to the control. This increase in phenolic compound content was also more marked, in Aouija (Fig 2D). The content of phenolic compounds increased under the effect of the combined stresses. The maximum increase was scored under severe stress (25% FC at 35°C) with an increase of 275.1%, followed by an increase of 198.9% under moderate stress (50% FC at 30°C) in comparison to that of the control treatments. The genotype Aouija had the highest content of phenolic compounds, with a fourfold increase compared to other genotypes under severe combined stress followed by the landrace Hedhba (357.7%) according to Tukey’s test (Fig 2D).

Besides, GPX activity was markedly stimulated under combined stress than under individual stresses (S1 Table). Indeed, GPX increased by 114.8% and 146.6% under moderate drought stress (50% FC) and severe stress (25% FC), respectively compared to the control (Fig 2E). The maximum GPX activity was observed for the two landraces Aouija (217.5%) and Hedhba (210.6%), against a minimum for Karim and Hmira (Fig 2E). The results showed that GPX increased significantly under heat stress. Thus, under 30°C, the average increase in GPX was 95.7% compared to the control with a maximum in Hedhba (181.5%) (Fig 2E). Under severe stress (35°C), the increase in GPX reached 131.4% compared to the control with a maximum increase in the two landraces Hedhba and Aouija, respectively with 217.3% and 186.1%, and a minimum in the improved genotype Biskri (71.8%) (Fig 2E). The GPX content tended to increase under the effect of combined stress with a maximum of 197.8% recorded under severe stress (25% FC at 35°C) against 142% under moderate stress (50% FC at 30°C). Our results showed that the genotype Aouija had the highest specific GPX activity (297.3%) followed by Hedhba (Fig 2E).

The increase in catalase activity under drought stress was used as an indicator of the redox state of the plant. Indeed, CAT is a constantly renewed enzyme in leaf cells, especially under stressful conditions [64]. The current study showed that catalase is significantly stimulated under drought stress, with a tendency to increase compared to the control. A higher CAT increase was observed under severe stress of 25% FC (157.4%), than under moderate stress (110.1%) compared to the control (Fig 2F). The highest increase in CAT activity was observed in Aouija (220.2%) under 25% FC. Under heat stress, the temperature of 30°C induced an increase in CAT of 91.5% compared to the control. The highest increase was observed for Aouija (207.1%). However, the genotype Karim had the lowest activity (Fig 2F). On the other hand, under severe stress (35°C), all the tested genotypes showed a higher significant increase of CAT than under moderate stress. In fact, under 35°C, the CAT increased on average by 115.2% compared to the control. The highest increase was observed in Aouija (Fig 2F). The enzymatic activity of CAT under the severe combination of drought and heat was more stimulated than under individual stresses (S1 Table). Thus, under (25% FC at 35°C), the average increase in CAT was 200.3% compared to the control with a maximum in the two landraces Aouija (280.9%) and Hedhba (232.6%), and a minimum in the improved genotype Karim (127.6%) (Fig 2F).

#### Relationships between screening traits for tolerance to drought, heat, and combined stress

Correlation analyses concerned morphological (APL), physiological (DM), and biochemical traits including H_2_O_2_ content, enzymatic antioxidants (GPX and CAT) and non-enzymatic antioxidants (phenolic compounds) under drought, heat, and combined stress were performed.

**Fig 3:**
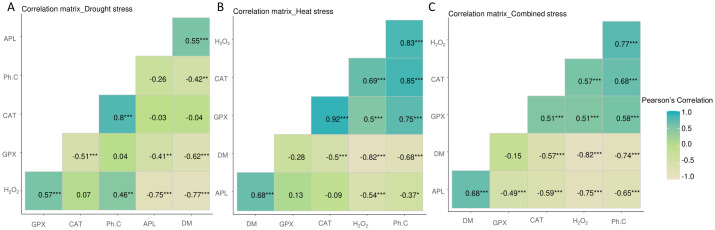
Graphical representation of the pearson correlations (α = 5%) of the measured parameters of five durum wheat genotypes under drought stress (100% FC, 50% FC, and 25% FC), heat stress (24°C, 30°C and 35°C) and combined stress (100% at 24°C, 50% FC at 30°C and 25% FC at 35°C). The beige coloring indicates the weakest correlations, and the turquoise coloring indicates the highest correlations. APL: Aerial part length, DM: Dry matter rate, H_2_O_2_: H_2_O_2_ content, phenolic compounds content (Ph.C), GPX: GPX activity, CAT: CAT activity. *, *P*<0.05; **, *P*<0.01; ***, *P*<0.001; NS: Not significant.

Under drought stress, correlation analysis (Fig 3A) highlighted the positive correlations between DM and APL (r = 0.55; *P* < 0.001), while DM showed a negative correlation with H_2_O_2_ (r = - 0.77; *P*<0.001) and GPX (r = - 0.62; *P* < 0.001). Similarly, negative correlations were highlighted between APL and H_2_O_2_ (r = - 0.75; *P* < 0.001). In addition, and as expected, H_2_O_2_ was positively correlated with GPX (r = 0.57; *P*<0.001) and with Ph.C (r = 0.46; *P* < 0.01). Also, a positive correlation between CAT and Ph.C (r = 0.8; *P* < 0.001) (Fig 3A).

Under heat stress, correlation analysis (Fig 3B) highlighted the positive correlations recorded between Ph.C and H_2_O_2_ (r = 0.83; *P* < 0.001), CAT (r = 0.85; *P* < 0.001), GPX (r = 0.75; *P* < 0.001). In addition, and as expected, H_2_O_2_ was positively correlated with GPX (r = 0.5; *P* < 0.001) and with CAT (r = 0.69; *P* < 0.001) with positive correlations between GPX and CAT (r = 0.92; *P* < 0.001) (Fig 3B). However, a negative correlation was recorded between CAT and DM (r = - 0.5; *P* < 0.001) (Fig 3B).

Correlation analysis under combined stresses (Fig 3C) highlighted positive correlations recorded between Ph.C and H_2_O_2_ (r = 0.77; *P* < 0.001), CAT (r = 0.68; *P* < 0.001), and GPX (r = 0.58; *P* < 0.001), while Ph.C showed a negative correlation with DM (r = -0.74; *P* < 0.001), and APL (r = -0.65; *P* < 0.001). In addition, H_2_O_2_ is negatively correlated with APL (r = - 0.75; *P* < 0.001) and DM (r = - 0.82; *P* < 0.001). However, positive correlations were recorded between H_2_O_2_ and CAT (r = 0.57; *P* < 0.001), and GPX (r = 0.51; *P* < 0.001) (Fig 3C). On the other hand, other negative correlations, recorded under combined stress and not under individual stresses (drought or heat stress), between APL and CAT (r = - 0.59; *P* < 0.001), and GPX (r = - 0.49; *P* < 0.001) (Fig 3C).

#### Principal component analysis (PCA)

The PCA was carried out to (i) reduce the dimension of the feature set, (ii) geometrically represent all the genotypes, and (iii) establish the links between the selection parameters tested and the different genotypes under the different stress levels.

**Fig 4:**
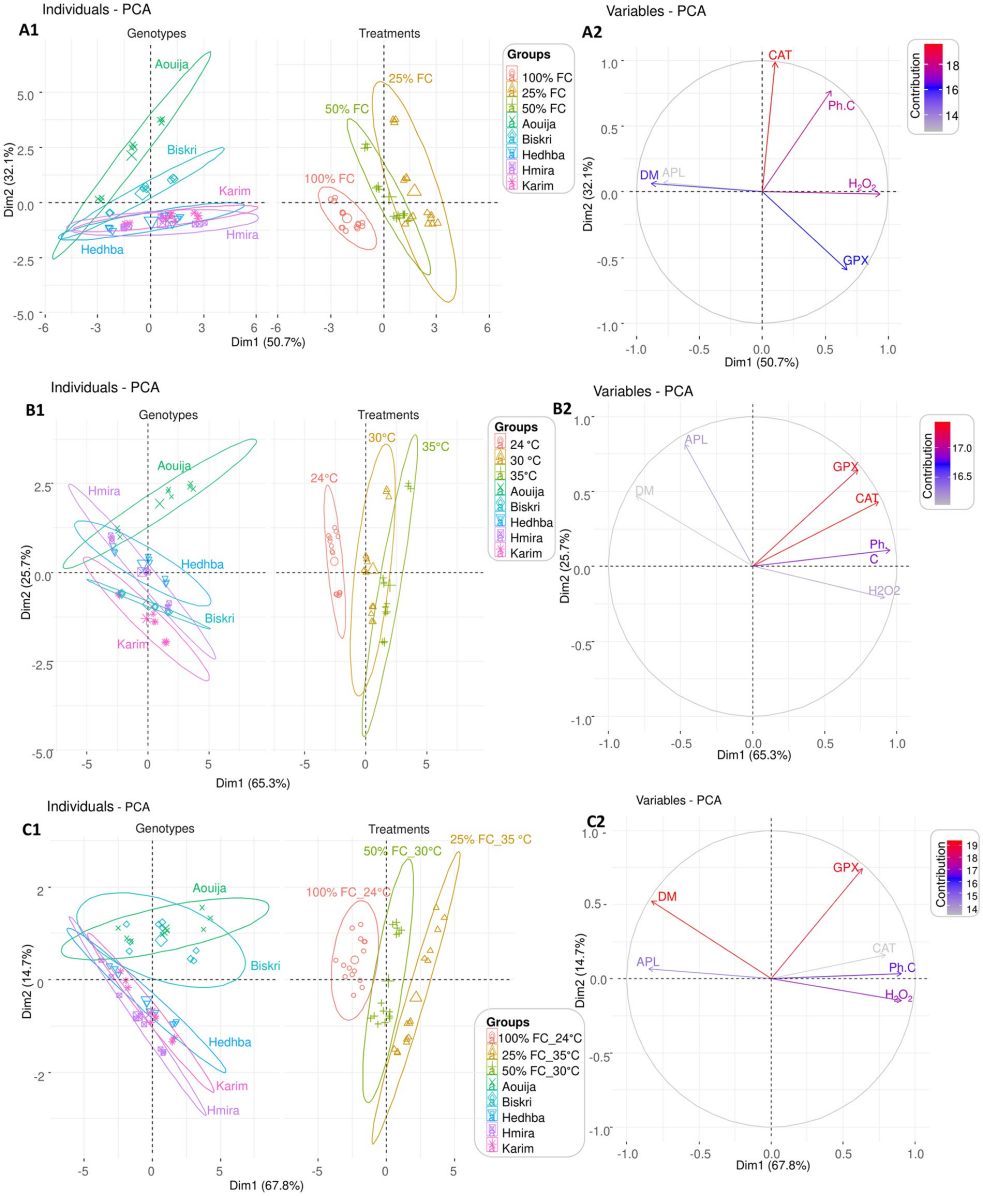
Principal component Analysis (PCA) of all the measured variables for the five genotypes under three studied stresses: (A) drought stress (100% FC, 50% FC, and 25% FC), (B) heat stress (24, 30, and 35°C), and (C) combined stress (100% FC at 24°C, 50%FC at 30°C, and 25% FC at 35°C). A1, B1, C1: PCA visualization of Individuals per genotype and treatment. Ellipses with 95% confidence were represented to allow clustering among genotypes and treatments. A2, B2, C2: Visualization of the contribution of each variable to the variance of the model with the direction of the variation. APL: Aerial part length, DM: Dry matter rate, H_2_O_2_: H_2_O_2_ content, Ph.C: phenolic compounds content, GPX: GPX activity, CAT: CAT activity.

Under water stress, the PCA explained 82.8% of the cumulative variance of all morpho-physiological (APL, DM) and biochemical (CAT, H_2_O_2_, GPX, and Ph.C) parameters of the five durum wheat genotypes (Fig 4A). This PCA revealed three groups of genotypes according to their degree of tolerance based on all the parameters measured (Fig 4A). The first group was constituted by the genotypes Karim, Hmira, and Hedhba. This group was characterized by high H_2_O_2_ content. The second group contained the genotype Biskri, and the third one contained Aouija. The last group was in direct opposition to the first one and was characterized by reduced H_2_O_2_ and the highest CAT activity and Ph.C. The result indicated that the genotype Aouija was potentially tolerant to drought by using oxidative stress mitigation mechanisms.

Under heat stress, PCA explained 91% of the cumulative variance (Fig 4B). Indeed, PCA revealed two groups of genotypes according to their degree of tolerance (Fig 4B). The first group was constituted by the genotypes Hedhba, Biskri, Karim, and Hmira. This group was characterized by high APL. The second group was constituted by the landrace Aouija in the opposite direction of the first one. The ellipses of 95% confidence level of each genotype indicated that there was a strong association between the variation of the GPX, CAT, and Ph.C and the landrace Aouija, and on the other hand, between the variation in H_2_O_2_ and the first group.

Besides under combined stress, PCA explained 82.5% of the cumulative variance (Fig 4C), providing three groups of genotypes (Fig 4C). The first group was constituted by the genotypes Karim, Hedhba, and Hmira and it was characterized by high H_2_O_2_ content. The second group contained the genotype Biskri, and the third one contained the genotype Aouija. A high GPX activity and Ph.C characterized the second and the third groups. On the other hand, DM and H_2_O_2_ content varied in opposite directions, indicating an independent behavior of both traits.

The ellipses of the three levels of treatments applied (control, moderate stress, and severe stress) were clearly separated for the three stresses (Fig 4A1–4C1). Under drought or/and heat stresses, the GPX, CAT, Ph.C, and H_2_O_2_ content were in the same direction. Those traits were stimulated under stressed treatments (moderate and severe conditions) (Fig 4A1–4C1). However, those traits were perpendicular to the DM and ALP’s vectors (Fig 4A2–4C2). Furthermore, as expected, DM and APL were favored under the control treatment (Fig 4A1–4C1).

**Fig 5:**
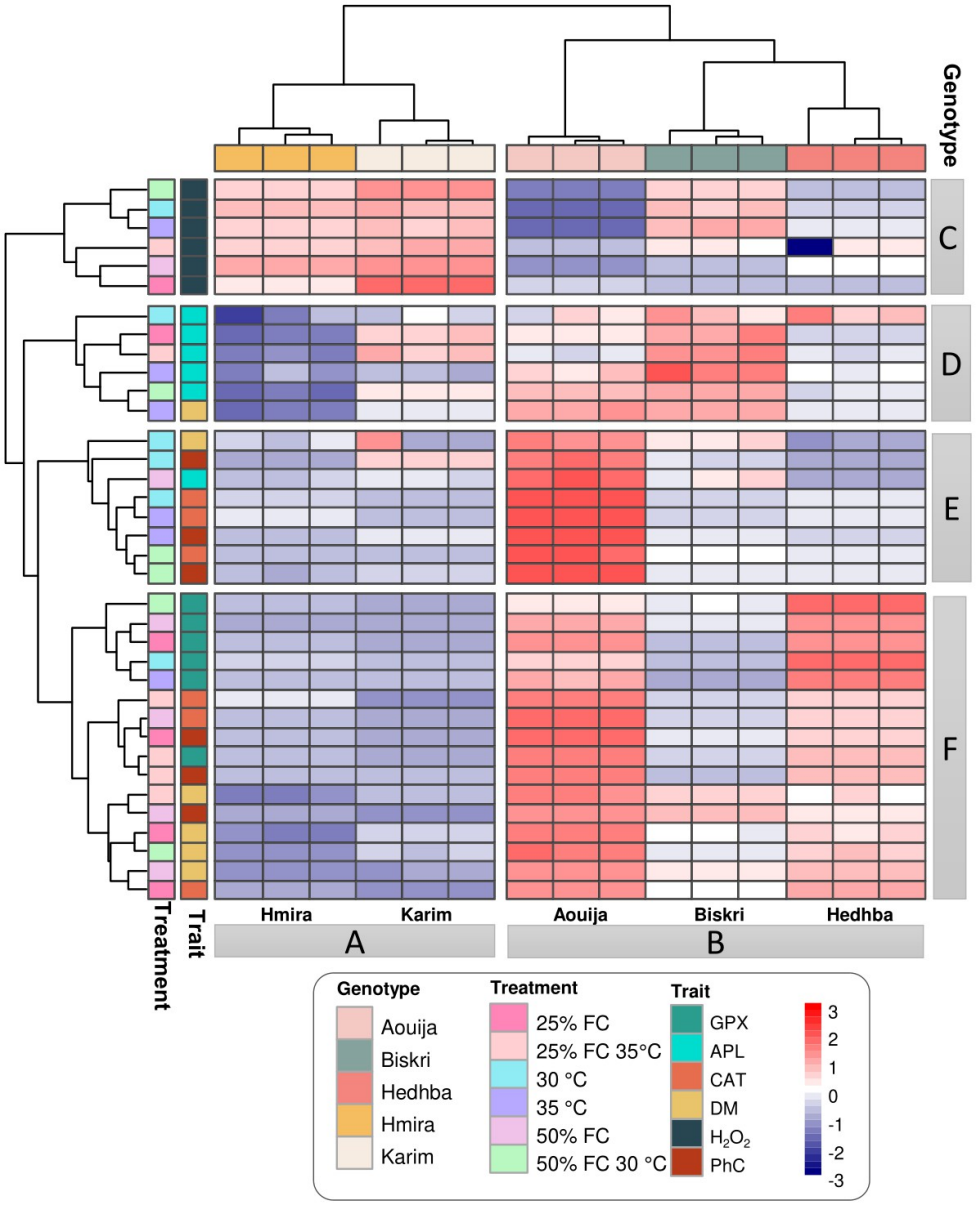
Heatmap representing hierarchical clustering of six measured traits across five durum wheat genotypes based on the significant rate change values in response to drought stress (50% FC, and 25% FC), heat stress (30°C and 35°C), and combined stress (50% FC at 30°C and 25% FC at 35°C) (Euclidean distance measure). A and B are the clusters based on the genotypes. C, D, E, and F are clusters based on the treatments and the assayed traits. The color bar depicts the gradient of rate change values in response to stress conditions. The different APL: Aerial part length, DM: Dry matter rate, H_2_O_2_: H_2_O_2_ content, Ph.C: Phenolic compounds content, GPX: GPX activity, CAT: CAT activity.

To present an overview of the measured traits and identify major clusters across the five genotypes under both control and stress conditions, hierarchical clustering was performed based on the significant rate change values by Euclidean distance measurement. The two main clusters (A and B) were considered for the studied genotypes (Fig 5). Cluster A included two genotypes Hmira and Karim which could be considered sensitive genotypes, showing higher accumulation of H_2_O_2_, but lower enzymatic (GPX and CAT) and non-enzymatic (Ph.C) antioxidants compared to cluster B. On the contrary, cluster B, including the three genotypes Aouija, Hedhba, and Biskri, which could be identified as the tolerant genotypes, showed lower H_2_O_2_ accumulation, mainly for the genotype Aouija, and higher DM and an active antioxidant defense system, chiefly for Aouija and Biskri (Fig 5). However, Hedhba had a similar bent as Aouija. Hence, the order of the top 3 most tolerant genotypes can be as follows; Aouija, Hedhba, and then Biskri.

## Discussion

This study investigated the genotypic variability of durum wheat under water, heat, and combined stresses. Plants respond to stress through morphological, physiological, and biochemical adaptations [65]. The great variability of response was noted for all the measured parameters. Indeed, the effect of stress depends on its degree, duration, stage of plant development, genotype, and interaction with the environment [66,67].

The results showed that a general reduction in APL and DM was observed under severe stress as water stress (25% FC) and heat (35°C). These results are also similar to those of [68–71], who described the harmful effect of these two stresses on the length of the aerial part of the plant. Nevertheless, these effects vary according to the adaptive strategy of each species or variety [72].

Our results showed that the genotypes Hmira and Karim were the most affected genotypes by drought, against a minimum growth for the genotype Hmira under heat stress. These results are in complete agreement with those obtained by [73]. On the other hand, both moderate (50% FC) and severe (25% FC) water stresses and moderate (30°C) and severe (35°C) heat stresses induced a drastic reduction in the rate of leaf dry matter (DM) in all genotypes. The DM is linearly related to stress intensity [74–76].

The evaluation of the biochemical components showed that water stress as well as heat stress induced an increased production of H_2_O_2_ as reported by several other works [77–81]. The maximum of H_2_O_2_ was noted under severe water stress (25% FC) as well as under heat stress (35°C). In response to both drought and heat stress, Hedhba and Aouija induced a weak accumulation of H_2_O_2_, this highlighted that these two genotypes could be potentially tolerant to oxidative damage.

To prevent and reduce oxidative damage caused by H_2_O_2_, plants have evolved an antioxidant defense mechanism to detoxify H_2_O_2_. This system can be classified as a combination of enzymatic factors including CAT and GPX activities [30,82,83], and non-enzymatic antioxidants as phenolic compounds [21,50,84]. Water and heat stress increased the specific activity of CAT in all the genotypes mainly in Aouija and Hedhba. CAT is an enzyme constantly renewed in leaf cells, especially under stress to eliminate H_2_O_2_ produced by photorespiration in peroxisomes [85]. According to [86,87], the activity of CAT increases in the leaves of cereals under water stress, which makes it a central marker in the protection against oxidative stress. The same trend was noted for GPX under water and heat stress, confirming several previous studies in durum wheat [88]. Indeed, the specific activity of GPX is considered an indicator of the redox state of plants [42]. Similarly, to cope with oxidative stress, the non-enzymatic antioxidant system is activated. Likewise, the stimulation of phenolic compounds was much more intensive under combined stress than under individual water and heat stress. Phenolic compounds are an indicator of the redox state of plants, they block auto-oxidation and the generation of active oxygen radicals, such as H_2_O_2_ [49,89]. In the case of the examined in the present study, a significant increase in the amount of total phenolics was observed, under drought or/and head stress conditions. The capacity of durum wheat to increase total phenolic accumulation has been directly linked to increased tolerance to numerous stressors, including drought and heat stress [50,90–92]. On the contrary, a reduction in total phenol concentration was reported in some previous research on the tolerance of wheat and maize genotypes to osmotic stress [93,94]. Such divergent findings from research investigating the accumulation of phenolics in stressful conditions may be related to the fact that their accumulation is dependent on the plant growth stage [95]. The findings of this study showed that the Aouija genotype had the highest content of phenolic compounds under water (25% FC) and heat (35°C) stresses. This genotype is also characterized by high enzymatic activities of CAT and GPX, thus having the highest antioxidant capacity compared to tested genotypes. The landrace genotypes Aouija and Hedhba were found to be more tolerant to water and heat stress as indicated by plant growth response, DM level, phenolic accumulation, and antioxidant activity.

In addition, the strong correlation between APL, DM, and antioxidants under the two stresses as previously reported [21] makes it possible to advance the opportunity to use those parameters as selection and reliable criteria to screen durum wheat drought and heat stress tolerance.

The same trends observed for individual water and heat stress were observed for combined stress. Thus, the lack of water accompanied by high temperature induced a significant decrease in APL [96]. This situation promotes more accelerated senescence and leaf abscission [97]. Similarly, the maximum reduction in DM was obtained under severe combined stress (25% FC at 35°C) mainly for Hmira and Karim. These results are in complete accordance with several other studies for the variety Karim [98–100]. Indeed, a reduction in the hydration of durum wheat induces a reduction in biomass [101–103]. Plant water status is highly dependent on thermal stability, and an excessive increase in heat causes dehydration of plant tissues, which limits the growth and development of plants [75]. The plant water retention ability is suggested to assess the resistance to heat stress and seems to be a good indicator of genotypic resistance to abiotic stresses [79]. The landraces Aouija, Biskri, and Hedhba showed the lowest reduction of APL and DM, which suggests that those landraces could be tolerant to combined stress despite the accumulation of stress effects. It is interesting to note, from PCA results, that, under both individual and combined stress, the landrace Aouija was the only genotype that kept the same tolerance to both abiotic stresses, which indicates that Aouija is the most stable genotype and could be considered as tolerant to drought or/and heat stresses. Furthermore, the landrace Aouija showed the same behavior under drought stress and combined drought and heat stresses in previous studies at the seedling stage [101,104]

Enzymatic (GPX and CAT) and non-enzymatic (Phenolic compounds) antioxidants are defense metabolites against the toxicity of ROS as H_2_O_2_ under abiotic stresses [105]. Under combined stress, the results showed an increase in these antioxidants in all genotypes. All five durum wheat genotypes reported in this study used the same strategies to mitigate the effect of combined stress [106]. Asserting the cumulative effect of individual stresses (drought and heat) which induce more remarkable damage. This accentuated effect of the combined stress is reflected by the weak growth and the strong reduction in the biomass. Therefore, abiotic stress at the vegetative stage can affect final production and yield [107]. This accentuated effect may be the result of the doubling of oxidative damage.

## Conclusion

The present study confirmed the durum wheat genotypic variability to mitigate abiotic stress at the juvenile growth stage. Water and heat stress cause profound morpho-physiological and biochemical changes in the various vegetative organs, such as in leaves. Indeed, the desiccation of the substrate induces a strong reduction in the dry matter of the aerial part of the plant observed in Hmira and Karim. However, a significant increase in enzymatic (GPX and CAT) and non-enzymatic (phenolic compounds) antioxidants was recorded mainly in the Aouija genotype followed by Hedhba genotype under the effects of water and heat stresses.

Under controlled conditions of the culture chamber, the results made under combined stress showed a decrease in the length of the aerial part, as well as the dry matter rate (DM). On the other hand, the combined stress induced an increase in enzymatic (GPX and CAT) and non-enzymatic (antioxidants) levels, mainly in Aouija and Hedhba, which suggests that those landraces could be tolerant to the toxicity of H_2_O_2_.

The results revealed that combined water and heat stresses had more significant harmful effects than individual stresses on durum wheat growth. The landraces genotypes Aouija, Hedhba, and Biskri showed tolerance aptitude to water and heat stress. Under combined stress, Aouija and Hedhba were the most adapted genotypes. According to tolerance stability, the landrace Aouija could be an interesting genetic source for breeding programs.

## Supporting information

S1 Table Means of all measured traits as the aerial part length (cm) (APL), phenolic compounds content (mg AGE/g Fresh Material (FM)) (Ph.C), Guaiacol peroxidases activity (mmole/min/mg proteins) (GPX), catalase activity (mmole/min/mg proteins) (CAT), dry matter rate (%) (DM), and the hydrogen peroxide content (nmol/g FM) (H_2_O_2_), for the five durum wheat genotypes conducted under three drought stress treatments: 100% FC, 50% FC, and 25% FC; three heat stress treatments: 24°C, 30°C, and 35°C; and three combined stress treatments: 100% FC_24°C, 50% FC_30°C, and 25% FC_35°C. Two-way ANOVA (up) and MANOVA (bottom) results are represented. Tukey’s test was performed to compare the treatment’s mean. *, *P* < 0.05; **, *P* < 0.01; ***, *P* < 0.001; NS: Not Significant.
(DOCX)

S1 Data Excel data (Excel file). (XLSX)
