# Supplementary material for: Genetic diversity of durum wheat (Triticum turgidum ssp. durum) to mitigate abiotic stress: Drought, heat, and their combination
Source: PLoS One. 2024 Apr 4;19(4):e0301018. doi: 10.1371/journal.pone.0301018 (PMC10994418; doi:10.1371/journal.pone.0301018)
Supplement: S1 Table — Two-way ANOVA (up) and MANOVA (bottom) results are represented. Tukey’s test was performed to compare the treatment’s mean. *, P < 0.05; **, P < 0.01; ***, P < 0.001; NS: Not Significant. (DOCX) [file pone.0301018.s001.docx]

**S1 Table.** Means of all measured traits as the aerial part length (cm) (APL), phenolic compounds content (mg AGE/g Fresh Material (FM)) (Ph.C), Guaiacol peroxidases activity (mmole/min/mg proteins) (GPX), catalase activity (mmole/min/mg proteins) (CAT), dry matter rate (%) (DM), and the hydrogen peroxide content (nmol/g FM) (H_2_O_2_), for the five durum wheat genotypes conducted under three drought stress treatments: 100% FC, 50% FC, and 25% FC; three heat stress treatments: 24°C, 30°C, and 35°C; and three combined stress treatments: 100% FC_24°C, 50% FC_30°C, and 25% FC_35°C. Two-way ANOVA (up) and MANOVA (bottom) results are represented. Tukey’s test was performed to compare the treatment’s mean. *, *P* < 0.05; **, *P* < 0.01; ***, *P* < 0.001; NS: Not Significant.

| **Genotypes (G)** | **Treatments (T)** | | **APL** | | | **DM** | | | **Ph.C** | | | **GPX** | | | **CAT** | | **H_2_O_2_** | | |
| --- | --- | --- | --- | --- | --- | --- | --- | --- | --- | --- | --- | --- | --- | --- | --- | --- | --- | --- | --- |
| **Aouija** | 24°C | | 53.7 | a | | 19.19 | a | | 0.04 | a | | 3.32 | a | | 1.67 | a | 0.07 | a | |
|  | 30°C | | 47.17 | b | | 17 | b | | 0.14 | b | | 7.31 | b | | 4.86 | b | 0.12 | b | |
|  | 35°C | | 44.58 | c | | 14.06 | c | | 0.21 | c | | 9.57 | c | | 5.29 | c | 0.13 | b | |
|  | 100% FC | | 49.33 | a | | 30.84 | a | | 0.08 | a | | 0.12 | a | | 0.07 | a | 0.04 | a | |
|  | 50% FC | | 41.83 | b | | 28.48 | ab | | 0.21 | b | | 0.26 | a | | 0.18 | b | 0.79 | b | |
|  | 25% FC | | 38.17 | b | | 25.24 | b | | 0.3 | c | | 0.3 | a | | 0.23 | c | 0.14 | b | |
|  | 100% FC_24°C | | 31.61 | | a | 21.74 | a | | 0.03 | | a | 1.55 | | a | 1.15 | a | 0.03 | | a |
|  | 50% FC _30°C | | 26.79 | | ab | 16.65 | b | | 0.11 | | b | 3.97 | | b | 3.61 | b | 0.08 | | b |
|  | 25% FC_35°C | | 22.83 | | b | 13.09 | c | | 0.15 | | c | 6.17 | | c | 4.4 | c | 0.11 | | c |
| **Hedhba** | 24°C | | 51.33 | | a | 19.42 | a | | 0.07 | | a | 1.31 | | a | 1.65 | a | 0.038 | | a |
|  | 30°C | | 45.8 | | b | 14.63 | b | | 0.11 | | b | 3.71 | | b | 2.9 | b | 0.09 | | b |
|  | 35°C | | 41.03 | | c | 11.42 | c | | 0.15 | | c | 4.17 | | c | 3.19 | c | 0.12 | | c |
|  | 100% FC | | 44.83 | | a | 35.01 | a | | 0.06 | | a | 1.54 | | a | 0.01 | a | 0.12 | | a |
|  | 50% FC | | 32 | | b | 20.98 | b | | 0.14 | | b | 3.98 | | b | 0.03 | b | 0.28 | | b |
|  | 25% FC | | 34.5 | | b | 19.08 | c | | 0.17 | | c | 4.79 | | c | 0.04 | b | 0.35 | | c |
|  | 100% FC_24°C **_** | | 35.69 | | a | 19 | a | | 0.03 | | a | 0.08 | | a | 1.15 | a | 0.03 | | a |
|  | 50% FC _30°C | | 27.04 | | b | 12.51 | b | | 0.08 | | b | 0.28 | | a | 2.76 | b | 0.09 | | b |
|  | 25% FC_35°C | | 25.77 | | b | 9.15 | b | | 0.13 | | c | 0.29 | | a | 3.82 | c | 0.14 | | c |
| **Hmira** | 24°C | | 50.53 | | a | 22.02 | a | | 0.07 | | a | 1.74 | | a | 1.67 | a | 0.04 | | a |
|  | 30°C | | 43.17 | | b | 17.15 | b | | 0.1 | | b | 2.86 | | a | 2.62 | b | 0.12 | | b |
|  | 35°C | | 38 | | c | 10.05 | c | | 0.13 | | b | 3.27 | | a | 3.35 | c | 0.13 | | c |
|  | 100% FC | | 44.17 | | a | 25.33 | a | | 0.07 | | a | 1.68 | | a | 0.02 | a | 0.10 | | a |
|  | 50% FC | | 35.83 | | b | 21.35 | b | | 0.14 | | b | 3.03 | | b | 0.03 | b | 0.3 | | b |
|  | 25% FC | | 32,83 | | b | 13.3 | c | | 0.15 | | c | 3.28 | | c | 0.04 | b | 0.37 | | c |
|  | 100% FC_24°C | | 37.67 | | a | 21.29 | a | | 0.03 | | a | 0.14 | | a | 0.07 | a | 0.04 | | a |
|  | 50% FC _30°C | | 24.42 | | bc | 10.06 | b | | 0.07 | | b | 0.27 | | a | 0.15 | a | 0.13 | | b |
|  | 25% FC_35°C | | 25.22 | | c | 6.83 | c | | 0.09 | | b | 0.33 | | a | 0.19 | a | 0.18 | | c |
| **Karim** | 24°C | | 39.5 | | a | 18.21 | a | | 0.05 | | a | 1.42 | | a | 1.75 | a | 0.04 | | a |
|  | 30°C | | 34.4 | | ab | 14.39 | b | | 0.13 | | b | 2.14 | | a | 2.55 | b | 0.12 | | b |
|  | 35°C | | 30.39 | | b | 10.88 | c | | 0.12 | | b | 2.6 | | a | 2.91 | c | 0.14 | | c |
|  | 100% FC | | 35.67 | | a | 29.52 | a | | 0.07 | | a | 1.74 | | a | 0.03 | a | 0.06 | | a |
|  | 50% FC | | 30 | | b | 21.14 | b | | 0.14 | | b | 3.25 | | b | 0.06 | b | 0.2 | | b |
|  | 25% FC | | 24.83 | | b | 17.97 | b | | 0.15 | | b | 3.62 | | b | 0.06 | c | 0.31 | | c |
|  | 100% FC_24°C | | 30.25 | | a | 18.63 | a | | 0.03 | | a | 0.12 | | a | 1.27 | a | 0.04 | | a |
|  | 50% FC _30°C | | 24.17 | | a | 10.05 | b | | 0.08 | | b | 0.22 | | a | 2.8 | b | 0.15 | | b |
|  | 25% FC_35°C | | 23.64 | | a | 7.17 | b | | 0.08 | | b | 0.27 | | a | 2.88 | c | 0.2 | | c |
| **Biskri** | 24°C | | 38.7 | | a | 17.98 | a | | 0.06 | | a | 2.14 | | a | 1.53 | a | 0.04 | | a |
|  | 30°C | | 34.43 | | b | 14.88 | a | | 0.13 | | b | 3.3 | | ab | 2.51 | b | 0.11 | | b |
|  | 35°C | | 33.83 | | b | 12.95 | b | | 0.16 | | c | 3.67 | | b | 2.72 | c | 0.14 | | c |
|  | 100% FC | | 37.17 | | a | 36.08 | a | | 0.05 | | a | 0.12 | | a | 0.05 | a | 0.1 | | a |
|  | 50% FC | | 31 | | b | 28.48 | b | | 0.13 | | b | 0.24 | | a | 0.1 | b | 0.201 | | b |
|  | 25% FC | | 27.5 | | b | 21.42 | c | | 0.14 | | b | 0.25 | | a | 0.11 | c | 0.293 | | c |
|  | 100% FC_24°C **_** | | 28.33 | | a | 22.36 | a | | 0.04 | | a | 1.46 | | a | 1.22 | a | 0.043 | | a |
|  | 50% FC _30°C | | 24.43 | | a | 12.95 | b | | 0.10 | | b | 3.4 | | b | 3.01 | b | 0.145 | | b |
|  | 25% FC_35°C **_** | | 23 | | a | 11.5 | c | | 0.11 | | c | 3.74 | | c | 3.27 | c | 0.184 | | c |
| **SE** | | **0.79** | | | | **0.67** | | **0.03** | | | | **0.1** | | | **0.08** | **0.004** | | | |
| **Treatments** | | | | | | | | | | | | | | | | | | | |
| **50% FC** | | | 18.5 | | d | 113.1 | e | | 24.1 | | d | 147.6 | | f | 135.7 | e | 109.1 | | e |
| **25% FC** | | | 22.8 | | b | 144 | b | | 38.1 | | bc | 254 | | b | 173.4 | d | 155.1 | | b |
| **30°C** | | | 12.3 | | e | 94.1 | f | | 19.4 | | e | 166.5 | | e | 129.7 | f | 89.8 | | f |
| **35°C** | | | 19.3 | | d | 129.2 | d | | 38 | | c | 213.7 | | d | 184.7 | c | 114.4 | | d |
| **50% FC_30°C** | | | 21.7 | | c | 139.9 | c | | 39.7 | | b | 219.9 | | c | 193.8 | b | 147.6 | | c |
| **25% FC_35°C** | | | 25.9 | | a | 195.2 | a | | 53.9 | | a | 338.8 | | a | 270.6 | a | 199.8 | | a |
| **MANOVA** | | | | | | | | | | | | | | | | | | | |
| **Genotypes (G)** |  | | *** | |  | *** |  | | ** | |  | *** | |  | *** |  | *** | |  |
| **Treatments (T)** |  | | *** | |  | *** |  | | *** | |  | *** | |  | *** |  | *** | |  |
| **G * T** |  | | *** | |  | *** |  | | * | |  | *** | |  | *** |  | * | |  |
